# Fecal microbiota composition and function are associated with anxiety and depression in patients with inflammatory bowel disease

**DOI:** 10.1371/journal.pone.0337941

**Published:** 2025-12-19

**Authors:** Zhan Wang, Minsi Zhou, Yan Dang, Xueping Huang, Chenyue Xu, Fang Xu, Xinyi Xu, Peng Li, Shutian Zhang, Haiyun Shi, Jing Wu

**Affiliations:** Department of Gastroenterology, Beijing Friendship Hospital, Capital Medical University, State Key Laboratory of Digestive Health, National Clinical Research Center for Digestive Diseases, Beijing, China; Texas A&M University College Station: Texas A&M University, UNITED STATES OF AMERICA

## Abstract

**Background and aims:**

Increasing prevalence of anxiety and depression are found in patients with inflammatory bowel disease (IBD). Altered gut microbiome may affect the brain, resulting in psychiatric symptoms. We aimed to analyze the feature of gut microbiota in IBD patients with anxiety or depression.

**Methods:**

Anxiety and depression symptoms were assessed by the Hospital Anxiety and Depression Scale (HADS). Stool samples were collected from IBD patients, and the 16S rRNA sequencing was used to detect fecal microbiota. Metabolites were detected by Liquid Chromatography-Mass Spectrometry (LC-MS).

**Results:**

Among the involved IBD patients (n = 59), 28.81% had anxiety and 33.90% had depression. Linear discriminant analysis Effect Size (LEfSe) (LDA > 3.0) revealed that 4 genera (*Klebsiella, Alloprevotella, Barnesiella, Bacillus*) were enriched, while *Sellimonas* was depleted in the anxiety group. Enrichment of 2 genera (*Ruminococcus, Barnesiella*) were found in the depression group. In the anxiety group, valine, leucine and isoleucine degradation was enriched in fecal microbiota, with upregulation of 3-methyl-2-oxobutanoic acid involved in the above pathway. In the depression group, butanoate metabolism was enriched in fecal microbiota, with alpha-ketoglutaric acid involved. Lysine degradation were enriched in fecal microbiota, with pipecolic acid involved. Primary bile acid biosynthesis was depleted in fecal microbiota, with glycocholic acid involved. Pearson correlation analysis revealed a positive correlation between *Alloprevotella* and 3-methyl-2-oxobutanoic acid in the anxiety group. In patients with both anxiety and depression, four genera (*Subdoligranulum, Alloprevotella, Christensenellaceae R-7 group, Barnesiella*) were positively correlated with alpha-ketoglutaric acid.

**Conclusion:**

In this study, the alteration of composition of fecal microbiota was identified, and differential genus associated with IBD patients with anxiety or depression or both were explored. Change in function of microbiota was also discovered by the detection of differential pathways and fecal metabolites, which were associated with IBD patients with anxiety or depression or both.

## Introduction

Inflammatory Bowel Disease (IBD) represents a group of intestinal disorders that cause chronic nonspecific inflammation of the digestive tract [1,2]. The rising tide of prevalence of IBD can be observed globally [3]. Occurrence of IBD in China has been increasing due to urbanization [3–6], receiving more attention in the country [5]. The intersection of IBD with mental disorders is increasingly recognized, as IBD patients are significantly more prone to experience psychiatric conditions such as anxiety and depression [7–10]. Incidence of anxiety and depression accumulates along with the rising prevalence of IBD. The average rate of both anxiety (19.1%) and depression (21.2%) symptoms in IBD patients was higher than those in other people [8]. IBD patients with comorbid anxiety and depression experience accelerated disease course, increased pharmacotherapy needs, and worsened prognostic outcomes [11–13].

Proposed mechanisms of IBD-related anxiety/depression include brain morphological changes [14,15], gut microbial dysbiosis [16], and genetic predisposition [7]. IBD-associated mood disorders arise from gut-brain axis dysregulation, featuring mutual exacerbation between intestinal inflammation and psychiatric symptoms [7,17]. The gut microbiota plays a certain role in the pathogenesis of IBD [18,19] as well as in the development of mental system disorders [20–22]. Moreover, various metabolic pathways—such as primary bile acid biosynthesis, vitamin digestion and absorption, and carbohydrate metabolism—have been implicated in IBD [23]. Key microbial metabolites, including bile acids, short-chain fatty acids (SCFAs), and tryptophan metabolites, are reported to contribute to both IBD-associated gut-brain axis dysfunction [24] and psychiatric conditions such as anxiety [25,26] and depression [27]. These evidence supports the hypothesis that fecal microbiota-derived metabolites may act as crucial mediators in the interplay between the fecal microbiota and both IBD and neuropsychiatric disorders [28]. However, further investigation is needed on the relationship between fecal microbiota, fecal metabolites, and the presence of anxiety or depression in individuals with IBD.

Therefore, in this study, we aimed to depict the feature of fecal microbiota and metabolites of IBD patients with anxiety or depression, preparing for further exploration of casual relationship between fecal microbiota and emotional disorders of IBD patients.

## Methods

### 1. Study design and population

This is a prospective study which was conducted at Beijing Friendship Hospital from 2023/2/1 to 2023/9/1. This study included patients with confirmed diagnosis of IBD, who met the following criteria: (1) age ≥ 18 years; (2) signed research informed consent. Patients with the following conditions were excluded: use of systemic corticosteroids, psychotropic drugs, or antibiotics in the past 3 months; previous psychiatric/neurological diseases; lactation/pregnancy. The gender, age of diagnosis, age of onset (AO), disease activity (Mayo clinical score for ulcerative colitis patients and Crohn’s disease activity index score for Crohn’s disease patients), course, disease type, whether IBD was newly diagnosed (newly diagnosed IBD), treatment, smoke, alcohol and BMI of the enrolled patients were collected. All patients provided written informed consent for participation in this study. The study protocol was approved by the Clinical Research Ethics Committee of the Beijing Friendship Hospital (2022-P2-292-02) and registered at National Clinical Medical Research Platform (Project No. MR-11-23-021147).

### 2. Assessment of anxiety and depression

Anxiety and depression symptoms in IBD patients were assessed using the Hospital Anxiety and Depression Scale (HADS). HADS is extensively used in psychiatric and medical patients [29–32] and has been applied on IBD patients in previous research [33]. It is a questionnaire consists of 14 items and is equally divided into 2 subscales: anxiety (HADS-A) and depression (HADS-D). Severity of symptoms are rated on a 4-point Likert scale and range from 0 to 3 [34]. Mostly, cutoff scores for subscales are: 0–7 = normal, 8–10 = mild, 11–15 = moderate, and 16 = severe [35]. In this study, symptoms of anxiety or depression were judged to exist when each subscale scored ≥8 points.

### 3. 16s rRNA targeted metagenomics analyses of fecal microbiota

Stool samples were collected and immediately stored at −80 °C. DNA was extracted from fecal samples using the TIANamp Stool DNA Kit (TIANGEN BIOTECH, Beijing, China). The V3–V4 region of the bacterial 16S rRNA gene was then amplified by polymerase chain reaction (PCR) with specific primers (CCTAYGGGRBGCASCAG and GGACTACNNGGGTATCTAAT) [36,37]. The PCR was conducted using 15 µL of Phusion® High-Fidelity PCR Master Mix (New England Biolabs), with forward and reverse primers at a concentration of 0.2 µM each, and approximately 10 ng of template DNA. The amplification products were first verified by electrophoresis on a 2% agarose gel. Qualified PCR products were then purified using magnetic beads, quantified by enzyme-labeling, and pooled in equimolar amounts based on their quantified concentrations.

The pooled library was subjected to a second round of agarose gel electrophoresis, during which the target fragments (<500 bp) were excised for further processing [37,38]. A sequencing library was constructed from the qualified PCR products and assessed using Qubit and real-time PCR for quantification, along with a bioanalyzer for size distribution. The pooled library was sequenced on an Illumina platform.

Bioinformatic processing was performed as follows: after removal of barcode and primer sequences, paired-end reads were merged using FLASH (v1.2.11, http://ccb.jhu.edu/software/FLASH/) to generate raw tags [39]. Quality filtering was conducted with FASTP (v0.23.1) to obtain high quality data called clean tags [40]. Chimera sequences were removed by comparison against the Silva database (https://www.arb-silva.de/), yielding effective tags (S1 Table in S1 File). The DADA2 module in QIIME2 was applied for denoising to generate amplicon sequence variants (ASVs) [41]. Taxonomic annotation was performed in QIIME2 (version 2022.02) based on the Silva database. Finally, the ASV absolute abundance table was rarefied to the minimum sequence count across samples to standardize sequencing depth. The adequacy of sequencing depth and diversity capture was confirmed by alpha rarefaction curves (S1 Fig in S1 File) and Good’s coverage estimates (S2 Fig in S1 File).

### 4. Fecal metabolomics analysis

To analyze fecal metabolites, non-targeted metabolomics analysis was performed on fecal samples. Liquid Chromatography-Mass Spectrometry (LC-MS) was used to detect the samples. The identified metabolites were annotated using the Kyoto Encyclopedia of Genes and Genomes (KEGG) (https://www.genome.jp/kegg/pathway.html), the Human Metabolome Database (HMDB) (https://hmdb.ca/metabolites), and the LIPIDMaps database (http://www.lipidmaps.org/).

### 5. Statistical analysis

The demographic factors and clinical parameters of this study were described and analyzed by IBM SPSS Statistics (Version 29.0.1.0). Continuous variables were presented as median (interquartile range) or mean (standard deviation) depending on whether the data were normally distributed. Categorical variables were summarized as numbers and percentages (%). Measurement data conforming to a normal distribution were analyzed using t-tests, while those with abnormal distribution were compared between groups using the Mann-Whitney U test. Categorical data were analyzed using chi-square tests.

Alpha diversity was represented by the Shannon index and compared between groups by Wilcox rank sum test. Beta diversity analysis was evaluated by the Bray-Curtis distance adjusted for age, sex, disease activity and BMI, calculated by PERMANOVA with the adonis2 function in the R vegan package, visualized by principal co-ordinates (PCoA) analysis via ade4 in R (Version 1.7) [42], further compared between groups by permutational multivariate analysis of variance (PERMANOVA) via vegan(Version 2.7) in R (Version 4.4.3). Alpha and beta diversity was both calculated using QIIME 2 and R. Stacked bar charts of relative abundance was performed by GraphPad Prism 10 (Version 10.0.3) to visualize the composition of microbiota. Differentially abundant taxa were evaluated by Linear discriminant analysis (LDA) and effect size (LEfSe) and DESeq2 via microeco (Version 1.14.0), mecodev (0.2.0) [43], and DESeq2 (1.46.0) [44] in R. The critical point of the logarithmic LDA score was 3.0, and P < 0.05 indicated statistical significance. Tax4Fun2 (Version 1.1.5) [45] was used to perform functional prediction analysis on the bacterial community and differential pathway analysis were performed by Weltch’s t-test in STAMP (Version 2.1.3) and multivariate linear models via Maaslin2 (version 1.15.1) [46]. Spearman correlation analysis performed by GraphPad Prism 10 (Version 10.0.3) was used to explore the correlation between the anxiety and depression scores of IBD patients and the abundance of differential microbiota. Figures in this article were produced by ggplot2 (Version 3.5.2) [47], extrafont (Version 0.19) [48], grid (Version 4.4.3) [49] and ComplexHeatmap (Version 2.23.1) [50,51] in R.

Multivariate statistical analysis of fecal metabolites was performed by metaX [52]. The default criteria for differential metabolite screening are VIP > 1, P < 0.05 and FC > 1.5. FDR-adjusted P values were calculated by dplyr (Version 1.1.4) in R [53]. Pearson correlation analysis was performed to investigate the association between differential microbiota and metabolites, which was performed by psych (Version 2.5.3) [54] in R. P < 0.05 indicated statistical significance in all the statistical and bioinformatical analysis above.

## Results

### 1. Demographical features of the study cohort

A total of 59 patients were involved in the study (median age, 38; male, 54.24%; female, 45.76%). The portion of remission was 54.24%, while 45.76% of them were active. There were 34 (57.63%) IBD patients without anxiety or depression (NonAD). We found 17 patients with HADS-A ≥ 8 points, which was considered as the anxiety group. Meanwhile, 20 patients with HADS-D ≥ 8 points were defined as the depression group. Moreover, 12 patients who scored both ≥8 on both HADS-A and HADS-D were defined as the group of IBD patients with both anxiety and depression (AD). Among 32 remission IBD patients, 8 were combined with anxiety, 9 were combined with depression. Among 27 active IBD patients, 9 were combined with anxiety, 11 were combined with depression. No statistical significance of age, sex, course, age of onset (AO), disease type, disease activity, Mayo score, treatment, whether IBD was newly diagnosed (newly diagnosed IBD), BMI and alcohol were discovered between NonAD and anxiety, NonAD and depression, NonAD and AD (Table 1). Therefore, no statistical differences in patient demographics and clinical characteristics were found after grouping by HADS-A and HADS-D scores in IBD patients.

**Table 1:** Baseline information of IBD patients with or without anxiety and/or depression.

Variables	NonAD(N = 34)	Anxiety (N = 17)	Value	P value	Depression (N = 20)	Value	P value	Anxiety and Depression (N = 12)	[n(%),x ± s, M(P25,P75)]	
									Value	P value
**Sex (n, %)**			<0.001	1.000		0.044	0.835		0.451	0.502
** Male**	(18,52.94)	(9,52.94)			10 (50.00)			5 (41.67)		
** Female**	(16,47.06)	(8,47.06)			10 (50.00)			7 (58.33)		
**Age at enrollment (median, range)**	36 (21 72 )	39 (23, 76 )	−1.050	0.294	49.55 ± 17.17	−1.963	0.050	53 (23.76)	−1.752	0.080
**Type (n, %)**			0.331	0.565		0.677	0.411		0.188	0.665
** UC**	(30,88.24)	(14,82.35)			16 (80.00)			10 (83.33)		
** CD**	(4,11.76)	(3,17.65)			4 (20.00)			2 (16.67)		
**Disease activity (n, %)**			0.354	0.552		0.597	0.440		1.804	0.179
** Activity**	(15,44.12)	(9,52.94)			11 (55.00)			8 (66.67)		
** Remission**	(19,55.88)	(8,47.06)			9 (45.00)			4 (33.33)		
**Course (month)**	47.50 (22.00,79.00)	49 (27, 126)	−0.450	0.653	29.5 (17.0, 100.0)	−0.197	0.844	42.5 (20.25,113.00)	−0.013	0.990
**Age at Onset (AO)**	32.50 (26 39 )	38.94 ± 13.47	−0.810	0.418	42.65 ± 15.97	−1.883	0.060	43.50 ± 13.19	−1.816	0.070
**Mayo score**	0.50 (0.00,5.25)	1.00 (0.00,3.25)	−0.321	0.749	1 (0,3)	−0.404	0.686	2.2 ± 2.10	−0.246	0.806
**Location of UC (n, %)**			0.378	0.828		1.129	0.569		1.397	0.497
** E1**	12 (35.29)	5 (29.41)			4 (20.00)			2 (16.67)		
** E2**	8 (23.53)	5 (29.41)			6 (30.00)			4 (33.33)		
** E3**	10 (29.41)	4 (23.53)			6 (30.00)			4 (33.33)		
**Location of CD (n, %)**			−	−		−	−		−	−
** L1**	1 (2.94)	0 (0.00)			0 (0.00)			0 (0.00)		
** L2**	0 (0.00)	0 (0.00)			1 (5.00)			0 (0.00)		
** L3**	3 (8.82)	2 (11.76)			2 (10.00)			1 (8.33)		
** L4**	0 (0.00)	1 (5.88)			1 (5.00)			1 (8.33)		
**Newly diagnosed IBD (n, %)**			−	−		−	−		−	−
** NO**	32 (94.12)	17 (100.00)			20 (100.00)			12 (100.00)		
** YES**	2 (5.88)	0 (0.00)			0 (0.00)			0 (0.00)		
**Treatment (n, %)**			1.695	0.428		1.583	0.453		1.695	0.428
**5-ASA**	25 (73.53)	12 (70.59)			15 (75.00)			9 (75.00)		
** Glucocorticoid (GSs)**	1 (2.94)	2 (11.76)			2 (10.00)			2 (16.67)		
** Biologics**	8 (23.53)	3 (17.65)			3 (15.00)			1 (8.33)		
**Smoke (n, %)**			7.650	0.006		2.67	0.102		5.436	0.020
** NO**	33 (97.06)	12 (70.59)			17 (85.00)			9 (75.00)		
** YES**	1 (2.94)	5 (29.41)			3 (15.00)			3 (25.00)		
**Alcohol (n, %)**			0.000	1.000		−	−		−	−
** NO**	32 (94.12)	13 (76.47)			20 (100.00)			12 (100.00)		
** YES**	2 (5.88)	1 (5.88)			0 (0.00)			0 (0.00)		
**BMI**	21.35 (19.72, 23.79)	23.26 ± 3.79	−1.219	0.223		−0.976	0.329	22.96 ± 3.51	−0.863	0.388
**HADS-A**	3 (1, 5)	11.47 ± 3.22	−5.798	**<0.001**	8 (6,12.5)	−6.121	**<0.001**	11.75 ± 3.67	−5.13	**<0.001**
**HADS-D**	2 (0,4)	10 ± 3.95	−5.499	**<0.001**	11.25 ± 3.02	−6.121	**<0.001**	11.67 ± 3.23	−5.139	**<0.001**
**HADS**	5 (1, 9)	21.35 ± 5.89	−5.759	**<0.001**	20.60 ± 5.83	−6.085	**<0.001**	23.42 ± 5.63	−5.119	**<0.001**

Non, IBD patients without anxiety or depression. 5-ASA, 5-Aminosalicylic acid. BMI, body mass index. HADS, Hospital Anxiety and Depression Scale. HADS-A, Hospital Anxiety and Depression Scale-Anxiety. HADS-D, Hospital Anxiety and Depression Scale-Depression. UC, ulcerative colitis. CD, Crohn’s disease. E1, proctitis. E2, left-sided colitis. E3, extensive colitis. L1, ileal. L2, colonic. L3, ileocolonic. L4, upper GI disease.

### 2. Richness and composition of fecal microbiota in IBD patients with anxiety or depression

**Fig 1:**
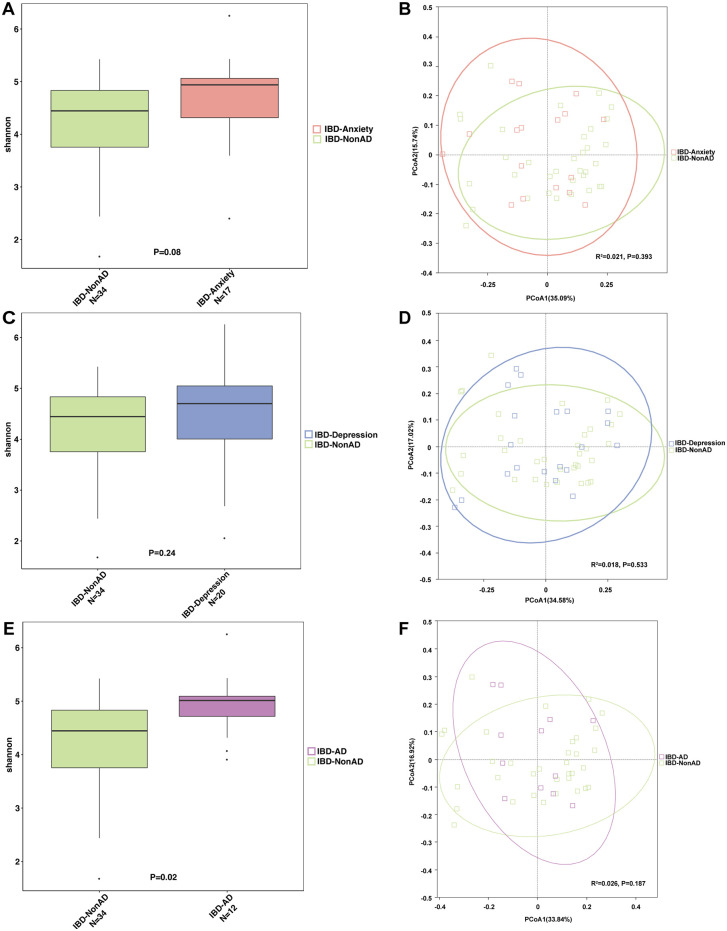
Bacterial diversity between IBD patients. A-B. Alpha diversity of anxiety **(A)**/depression **(C)**/anxiety and depression **(E)** in IBD patients was described with Shannon index. PCoA plot indicating beta diversity of anxiety **(B)**/depression **(D)**/ anxiety and depression **(F)**. IBD-Non, IBD patients without anxiety or depression. IBD-AD, IBD patients with both anxiety and depression.

Compared with IBD patients without anxiety or depression (NonAD), alpha diversity and beta diversity of fecal microbiota in IBD patients with anxiety (alpha diversity, P = 0.08, Fig 1A; beta diversity, R^2 ^= 0.021, P = 0.393, Fig 1B, S2a Table in S1 File), depression (alpha diversity, P = 0.24, Fig 1C; beta diversity, R^2 ^= 0.018, P = 0.533, Fig 1D, S2b Table in S1 File) did not reach statistical significance. Significant difference of alpha diversity was found in IBD patients with both anxiety and depression (AD), while no significance of beta diversity was achieved (alpha diversity, P = 0.02, Fig 1E; beta diversity, R^2 ^= 0.026, P = 0.187, Fig 1F, S2c Table in S1 File). In summary, significant alterations in microbial diversity were only observed as reduced alpha diversity in patients with both anxiety and depression, but not in those with either condition alone.

### 3. Taxonomical diversity and difference of fecal microbiota in IBD patients with anxiety or depression

**Fig 2:**
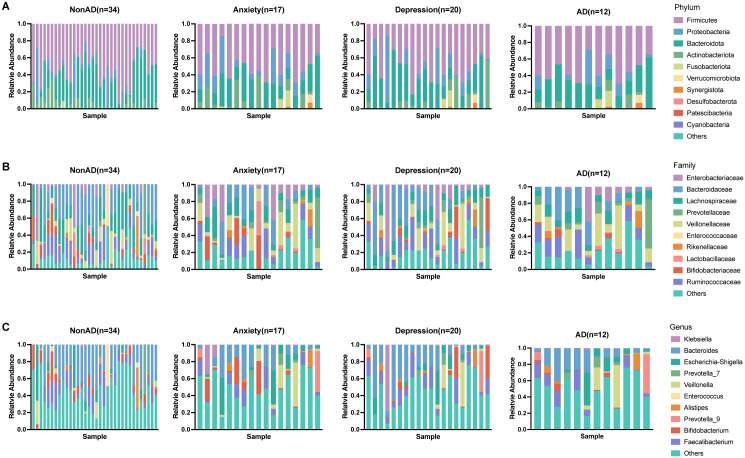
Community composition shown by relative abundance of prevalent (A) phyla, (B) families and (C) genus. NonAD, IBD patients without anxiety or depression symptoms. AD, IBD patients with both anxiety and depression.

The dominant phyla present in the fecal samples among all the groups were *Firmicutes* (49.8 ± 18.3%), *Bacteroidota* (28.5 ± 21.0%), and *Proteobacteria* (11.6 ± 16.9%) (Fig 2A). *Bacteroidaceae* (22.8 ± 20.2%) was the dominant family in the study (Fig 2B). *Bacteroides* (14.8 ± 13.3%) was the most prevalent genus in IBD patients (Fig 2C). In summary, according to the figure of community composition, 3 phyla (*Firmicute*s, *Bacteroidota, Proteobacteria*), family *Bacteroidaceae,* genus *Bacteroides* were the dominant taxon in the fecal samples.

**Fig 3:**
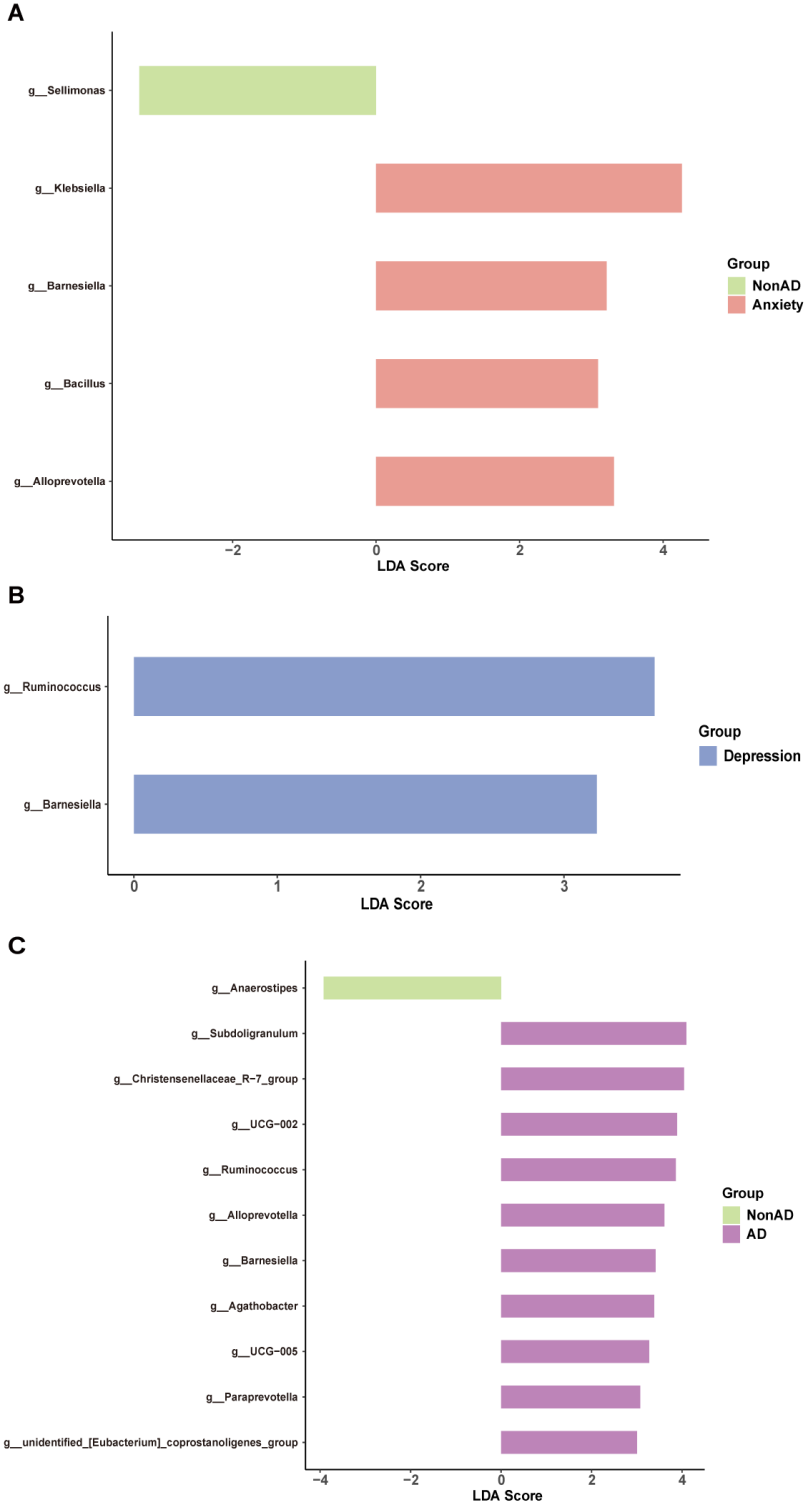
LEfSe bar plot of differential genera in NonAD VS anxiety (A), NonAD VS depression (B), NonAD VS anxiety and depression (C). NonAD, IBD patients without anxiety or depression symptoms. AD, IBD patients with both anxiety and depression.

In genus level, compared with patients without anxiety or depression, LEfSe revealed that the relative abundance of 4 genera (*Klebsiella, Alloprevotella, Barnesiella, Bacillus*) were upregulated in IBD patients with anxiety, while the relative abundance of *Sellimonas* was depleted in the anxiety group (Fig 3A, S3 Fig in S1 File). As for IBD patients with depression in this study, the relative abundance of 2 genera were upregulated (*Ruminococcus, Barnesiella*) (Fig 3B, S4 Fig in S1 File). Enrichment of 10 genera (*Subdoligranulum, Christensenellaceae_R-7_group, UCG-002, Ruminococcus, Alloprevotella, Barnesiella, Agathobacter, UCG-005, Paraprevotella, unidentified_[Eubacterium]_coprostanoligenes_group*) and depletion of genus *Anaerostipes* were discovered in IBD patients with both anxiety and depression (Fig 3C, S5 Fig in S1 File). DESeq2 was also performed to search for differential ASVs between different groups. ASVs of *Klebsiella* and *Sellimonas* in IBD patients with anxiety (S3a Table in S1 File), *Ruminococcus* in IBD patients with depression (S3b Table in S1 File), *Christensenellaceae_R-7_group, UCG-002, Ruminococcus, Agathobacter, UCG-005, Paraprevotella, unidentified_[Eubacterium]_coprostanoligenes_group, Anaerostipes* in IBD patients with both anxiety and depression (S3c Table in S1 File) were significantly changed, which was consistent with results found by LEfSe. These results collectively demonstrated that distinct fecal microbiota signatures were associated with anxiety, depression, and their co-occurrence in IBD patients.

**Fig 4:**
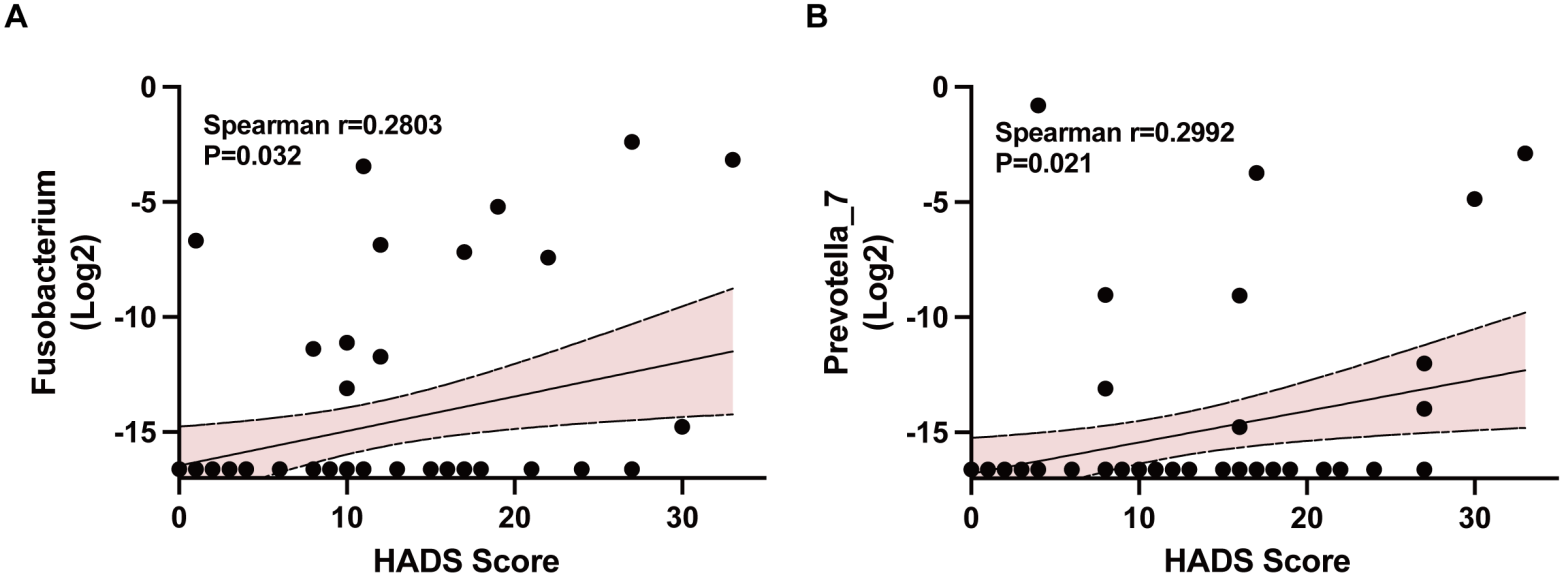
Spearman Correlation analysis between HADS and Fusobacterium (A)/Prevotella_7 (B). The X-axis is the score of HADS, and Y-axis is the log2 genus relative abundance. Area in represents a positive correlation trend, and each point represents the ASVs included in the Spearman correlation analysis of the genus and HADS. *Because the relative abundance of bacteria was not detected in some fecal samples, we used the pseudo count method to add 0.00001 to the original relative abundance of each sample and then normalized the logarithm of the relative abundance value before performing correlation analysis.

We further explored Spearman correlation analysis between microbiota abundance at the genus level and HADS, HADS-A, HADS-D score. Significant positive correlation was found between the relative abundance of *Fusobacterium* and HADS score (r = 0.2803, P = 0.032, Fig 4A), *Prevotella_7* and HADS score (r = 0.2992, P = 0.021) (Fig 4B). DESeq2 also showed significance of *Fusobacterium* between NonAD and AD patients (P = 0.037, FDR-adjusted, S3c Table in S1 File). Bar plots of relative abundance of *Fusobacterium* and *Prevotella_7* between groups were shown in S6 Fig in S1 File. This analysis identified *Fusobacterium* and *Prevotella_7* as specific genera whose relative abundance was positively linked with the severity of psychological symptoms.

**Fig 5:**
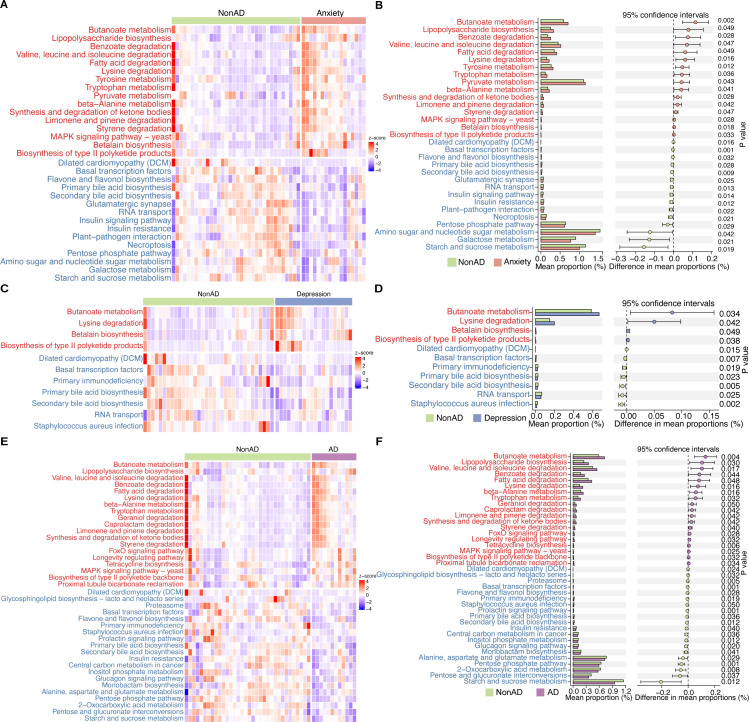
Relative abundance heatmap of Tax4Fun2 functional annotations (Level 3) in anxiety (A)/depression (C)/anxiety and depression (E). The horizontal ordinate indicates grouping of samples. The vertical ordinate indicates function annotation. The color scale represents the z-score of the relative abundance of each row after normalization. Differential analysis of the relative abundance of Tax4Fun2 functional annotations (Level 3) in anxiety **(B)**/depression **(D)**/anxiety and depression **(F)**. Non, IBD patients without anxiety or depression. AD, IBD patients with both anxiety and depression.

Meanwhile, functional prediction of gut microbiota was performed via Tax4Fun2. Significantly increasing functional relative abundance of 16 pathways, including tryptophan metabolism (P = 0.036), valine, leucine and isoleucine degradation (P = 0.046), and decreasing of 15 pathways were observed in IBD patients with anxiety (Fig 5A and 5B, S4a Table in S1 File). For IBD patients with depression, enrichment of 4 pathways, including butanoate metabolism (P = 0.034), lysine degradation (P = 0.041), and depletion of 7 pathways including primary bile acid biosynthesis (P = 0.023) were observed (Fig 5C and 5D, S4b Table in S1 File). Compared with IBD patients without anxiety or depression, IBD patients with both anxiety and depression had 19 enriched pathways including valine, leucine and isoleucine degradation (P = 0.017), lysine degradation (P = 0.015) and tryptophan metabolism (P = 0.032), as well as 20 depleted pathways including 2-oxocarboxylic acid metabolism (P = 0.007), pentose and glucuronate interconversions (P = 0.037) and primary bile acid biosynthesis (P = 0.036) (Fig 5E and 5F, S4c Table in S1 File). All pathways related with differential fecal metabolites remained significance in the multivariate linear models (S7-S9 Figs in S1 File). In conclusion, these functional alterations suggested that shifts in key microbial metabolic pathways, particularly in amino acid and bile acid metabolism, were the feature of IBD patients with anxiety and/or depression in this study.

### 4. Associations between the differential fecal microbiota and fecal metabolites in IBD patients

In IBD patients with anxiety, we found 25 significantly upregulated and 66 downregulated fecal metabolites (S5 Table in S1 File). In IBD patients with depression, we discovered 38 significantly upregulated and 104 downregulated fecal metabolites (S6 Table in S1 File). IBD patients with both anxiety and depression had 107 fecal metabolites upregulated and 45 fecal metabolites downregulated (S7 Table in S1 File). Together, these results demonstrate that the presence of anxiety, depression, or both in IBD patients was associated with specific alterations in the fecal metabolites.

After annotating the above differential metabolites according to KEGG, we found that several metabolites were involved in the pathways that reached statistical significance in the microbiota. In IBD patients with anxiety, 3-methyl-2-oxobutanoic acid involved in valine, leucine and isoleucine degradation was upregulated (P = 0.044). In IBD patients with depression, alpha-ketoglutaric acid involved in butanoate metabolism (P = 0.048), pipecolic acid involved in lysine degradation (P = 0.048), glycocholic acid involved in primary bile acid biosynthesis (P = 0.037) were all downregulated. In IBD patients with both anxiety and depression, alpha-ketoglutaric acid involved in pentose and glucuronate interconversions (P = 0.043), taurochenodeoxycholic acid involved in primary bile acid biosynthesis (P = 0.024), 2-keto-4-methylthiobutyric acid involved in 2-oxocarboxylic acid metabolism (P = 0.049) were downregulated. N6, N6, N6-trimethyl-L-lysine involved in lysine degradation (P = 0.043), indole-3-acetic acid involved in tryptophan metabolism (P = 0.021) were upregulated. This indicated that the functional pathway alteration predicted by microbiota analysis were reflected by corresponding changes in specific host and microbial metabolites.

To explore the relationship between differential genus and differential metabolites mentioned in the previous paragraph, we further conducted Pearson correlation analysis between them. In IBD patients with anxiety (Table 2), we found a significant positive correlation between *Alloprevotella* and 3-methyl-2-oxobutanoic acid (r = 0.775, p < 0.001). No statistically significant correlation of differential genus and metabolites were found in IBD patients with depression (Table 3). In AD group (Table 4), we found five pairs of differential genus and metabolites with statistically significant differences: *Subdoligranulum* and alpha-Ketoglutaric acid (r = 0.530, P < 0.001), *Alloprevotella* and alpha-ketoglutaric acid (r = 0.564, P < 0.001), *Alloprevotella* and 2-keto-4-methylthiobutyric acid (r = 0.829, P < 0.001), *Christensenellaceae_R-7_group* and alpha-ketoglutaric acid (r = 0.366, P = 0.012), *Barnesiella* and alpha-ketoglutaric acid (r = 0.599, P < 0.001). These correlations provided mechanistic insights by linking specific anxiety- and depression-associated genera to the dysregulation of key metabolites. These significant correlations supported a hypothesized interaction between the differential fecal microbiota and the dysregulated metabolites in IBD patients with anxiety, depression, or both.

**Table 2:** Pearson correlation between differential genus and metabolites in IBD patients with anxiety.

Genus	3-methyl-2-oxobutanoic acid (r)	P value
*Prevotella_7*	0.086	0.743
*Sellimonas*	−0.216	0.406
*Barnesiella*	0.039	0.882
*Paraprevotella*	−0.094	0.721
*Alloprevotella*	0.925	**<0.001**
*Klebsiella*	−0.007	0.980

r, Pearson correlation coefficient.

**Table 3:** Pearson correlation between differential genus and metabolites in IBD patients with depression.

Genus	Alpha-ketoglutaric acid (r)	P value	Pipecolic acid (r)	P value	Glycocholic acid (r)	P value
*Ruminococcus*	−0.029	0.834	0.205	0.137	0.193	0.162
*Barnesiella*	0.100	0.473	−0.037	0.789	−0.045	0.746

r, Pearson correlation coefficient.

**Table 4:** Pearson correlation between differential genus and metabolites in IBD patients with depression and depression.

Genus	Indole-3-acetic acid (r)	P value	Taurocheno-deoxycholic acid (r)	P value	Alpha-Ketoglutaric acid (r)	P value	N6,N6,N6-Trimethyl-L-lysine (r)	P value	2-Keto-4-methylthiobutyric acid (r)	P value
*Anaerostipes*	−0.215	0.152	−0.043	0.778	−0.149	0.324	0.136	0.367	−0.105	0.485
*Agathobacter*	0.091	0.549	−0.052	0.734	0.070	0.645	−0.094	0.534	0.081	0.595
*Subdoligranulum*	0.007	0.963	−0.085	0.573	0.530	**<0.001**	−0.202	0.178	0.263	0.077
*UCG-002*	0.154	0.305	−0.066	0.662	0.049	0.747	−0.039	0.798	−0.034	0.821
*Ruminococcus*	0.106	0.482	−0.065	0.669	0.112	0.460	−0.062	0.684	0.035	0.815
*Alloprevotella*	0.087	0.566	−0.034	0.821	0.564	**<0.001**	−0.097	0.520	0.829	**<0.001**
*Christensenellaceae_R-7_group*	0.069	0.648	−0.068	0.652	0.366	**0.012**	−0.187	0.213	0.158	0.295
*Paraprevotella*	−0.003	0.983	−0.032	0.834	0.125	0.408	−0.103	0.496	0.112	0.458
*UCG-005*	0.144	0.341	−0.044	0.773	0.020	0.897	−0.109	0.469	−0.044	0.772
*Barnesiella*	0.112	0.459	−0.048	0.751	0.599	**<0.001**	−0.028	0.854	0.272	0.067
*unidentified_[Eubacterium]_coprostanoligenes_group*	0.010	0.945	−0.044	0.770	0.287	0.053	−0.139	0.357	0.150	0.321

r, Pearson correlation coefficient.

## Discussion

This study identified the alteration of fecal microbiota and metabolites in IBD patients with anxiety, depression and both. Compared with IBD patients without anxiety or depression, we discovered enrichment of 4 genera, 31 KEGG predicted metabolic pathways and 91 differential fecal metabolites. Compared with IBD patients without anxiety or depression, we discovered enrichment of 2 genera, 11 KEGG predicted pathways and 142 differential fecal metabolites in IBD patients with depression. Compared with IBD patients without anxiety or depression, we discovered enrichment of 10 genera, 39 KEGG predicted pathways, 152 differential fecal metabolites in IBD patients with anxiety and depression.

In the anxiety group, we identified enriched genera including *Klebsiella*, *Alloprevotella*, *Barnesiella*, and *Bacillus*, alongside an upregulation of the valine, leucine, and isoleucine degradation pathway and its associated metabolite, 3-methyl-2-oxobutanoic acid. Our finding of *Klebsiella* enrichment is consistent with its previously reported association with anxiety [55] and its known role in IBD [56,57]. The enrichment of *Alloprevotella* aligns with studies reporting its increase in patients with major depressive disorder [58,59] and in mouse models of anxiety-like behavior [60]. This genus has also been frequently associated with states of chronic intestinal inflammation [61,62] and infection [63], with studies noting a decrease in its relative abundance following successful treatment of ulcerative colitis [64,65]. One study reported that stress-sensitive mice exhibited higher levels of *Alloprevotella* alongside enhanced expression of IBD-related genes and reduced hippocampal synaptic plasticity compared to stress-resistant mice [66], suggesting a potential pathway linking this genus to brain function. The upregulated metabolite 3-methyl-2-oxobutanoic acid, a branched-chain keto acid [67,68] and precursor to short-chain fatty acids (SCFAs) [69,70], has been previously noted to decrease with the amelioration of colitis symptoms in mice [69]. A positive correlation between *Alloprevotella* and 3-methyl-2-oxobutanoic acid was found among individuals in the anxiety group. While 3-methyl-2-oxobutanoic acid is a known precursor of SCFAs, and *Alloprevotella* may contribute to SCFA production [71–73], the direct interaction between them is not established. The relationship between this genus and metabolite, and their specific roles in anxiety/depression, remain unexplored and require further investigation.

In the depression group, we observed an enrichment of *Ruminococcus* and *Barnesiella*, a depletion of the primary bile acid biosynthesis pathway, and a downregulation of glycocholic acid compared to NonAD group. The enrichment of *Barnesiella* in our study is supported by its reported increase in Chinese patients with first-episode depression [74], in other depression cohorts from Europe and Asia [75], and in post-stroke depression patients [76]. Similarly, the association we found for *Ruminococcus* is consistent with previous studies linking this genus to depression [77,78]. *Ruminococcus* has been implicated in the production of pro-inflammatory polysaccharides [79], which may activate NF-κB signaling and potentially contribute to neuroinflammation [80]. Furthermore, *Ruminococcus* has been documented to participate in bile acid metabolism [81,82], which is relevant to our finding of a downregulated primary bile acid pathway. The observed downregulation of glycocholic acid (a major component of animal bile [83]) in our study aligns with a previous animal study that reported an elevation of this bile acid and the relief of depression-like behaviors in mice [84]. This convergence suggests that modulation of bile acid metabolism may be a significant facet of the gut-brain axis in depression.

In the AD group, we also discovered enrichment of *Alloprevotella*, *Ruminococcus*, *Barnesiella*, *Agathobacter*, *Paraprevotella*, *Subdoligranulum*, downregulation of pentose and glucuronate interconversions, upregulation of butanoate metabolism, down regulation of alpha-ketoglutaric acid compared to NonAD group. Association between anxiety/depression and *Alloprevotella*, *Ruminococcus*, *Barnesiella* has been discussed above. Our findings regarding *Agathobacter* [85] and *Christensenellaceae R-7 group* [86] are consistent with studies reporting their enrichment in individuals with depressive symptoms. Previous studies have also shown that *Paraprevotella* was positively correlated with anxiety [87,88], depression [89,90]. Higher relative abundance of *Subdoligranulum* was found to be related with depression [91,92], Generalized Anxiety Disorder-7 scores [93]. A study of patients with comorbid anxiety and acute coronary syndrome reported a similar decrease in pentose and glucuronate interconversions [94]. The upregulation of butanoate metabolism observed in our cohort aligns with a previous study, which reported an enhancement of this pathway in mice following a loss of social dominance, a condition reported to induce depression [95]. Furthermore, the downregulation of alpha-ketoglutaric acid in our patients is supported by a study showing reduced concentrations of it in the colon of mice with depression-like behaviors [96]. Furthermore, elevated oxidative stress under conditions of anxiety and depression [97] may lead to increased consumption of alpha-ketoglutaric acid [98], given its role as a key metabolic intermediate. In our study, alpha-ketoglutaric acid involved in butanoate metabolism and pentose and glucuronate interconversions showed significant positive correlations with several genera, including *Subdoligranulum, Christensenellaceae R-7 group, Alloprevotella*, and *Barnesiella*. However, evidence on mechanism indicating interplay between alpha-ketoglutaric acid and those genera are limited. As we can see, the reduction in fecal alpha-ketoglutaric acid may be influenced by multiple factors, including gut microbiota composition and host metabolic state. Further investigations are needed to explore its specific mechanism.

This study offers several strengths. First, by focusing specifically on patients with IBD, we aim to draw clinical attention to the psychological well-being of this population, further appealing for integrating psychological screening and mental health care into the standard management protocol for IBD patients. Second, we placed particular emphasis on the role of gut microbiota and fecal metabolites, thereby offering a more comprehensive and intuitive understanding of microbial involvement in IBD patients with anxiety and/or depression. Third, a variety of statistical analysis methods including LEfSe, DESeq2, Pearson correlation analysis, combined with functional prediction analysis was used to reveal the complex relationship between the microbiome and metabolites. Additionally, this real-world study documents the gut microbiota and metabolic features in the Chinese IBD population with anxiety and/or depression, offering insights and a reference basis for related research in Asian populations. Furthermore, by integrating fecal metabolite analysis and metabolic pathway investigation with microbial profiling, we reveal more specific interactions between gut microbiota and metabolites through metabolic pathways, offering specific, potential hypotheses for future mechanistic studies.

This study has several limitations. Firstly, the 16S rRNA sequencing used in this study provides only genus-level taxonomic resolution. Future research should employ higher-resolution techniques (e.g., shotgun metagenomics) to identify species-level microbial changes. As a result, reliability of functional prediction based on 16S rRNA sequencing may not be sufficient. More direct methods like meta transcriptomics would better characterize microbial metabolic pathways. Second, the cross-sectional design limits our ability to establish causal relationships between gut microbiota alterations and psychiatric symptoms in IBD. Longitudinal studies tracking microbial dynamics alongside symptom progression are needed to elucidate potential causative mechanisms underlying the gut-brain axis interactions in this population. Third, the relatively small sample size may have limited our statistical power, especially for sub-group analysis based on disease activity. Large-scale studies with stratification of disease activity would help reduce heterogeneity and provide more robust comparative analyses. The inclusion of healthy control groups would be particularly valuable for distinguishing IBD-specific microbial signatures from those associated with psychiatric conditions alone. Additionally, diet habits should also be included in the future due to its influence on microbiota. Besides, as activity is influential in IBD patients’ life quality, more analysis between disease activity and anxiety, depression should be conducted. Despite these limitations, our findings provide clinically relevant insights into the associations between gut microbiota composition, metabolic profiles, and psychiatric symptoms in IBD patients. These real-world data contribute to the growing evidence base supporting the gut-brain axis in IBD and may inform future therapeutic strategies targeting the microbiome for managing psychiatric comorbidities in this population and provide new directions for further studying how the brain-gut axis contributes to the interaction between IBD and psychological symptoms.

## Conclusions

Compared with IBD patients without anxiety and depression, patients with anxiety and depression had significant changes in fecal microbiota and metabolites to varying degrees. Differential genus associated with IBD patients with anxiety, depression or both was explored. Change in function of microbiota was also discovered by the detection of differential pathways and fecal metabolites.

## Supporting information

S1 File Supporting figures and tables. (ZIP)
