# Supplementary material for: Fecal microbiota composition and function are associated with anxiety and depression in patients with inflammatory bowel disease
Source: PLoS One. 2025 Dec 19;20(12):e0337941. doi: 10.1371/journal.pone.0337941 (PMC12716764; doi:10.1371/journal.pone.0337941)
Supplement: S1 File — (ZIP) [file pone.0337941.s001.zip › supplementary materials/Captions of supporting figures and tables.docx]

**Captions of supporting figures and tables**

Fecal Microbiota Composition and Function Are Associated with Anxiety and Depression in Patients with Inflammatory Bowel Disease

Zhan Wang^¶^, Minsi Zhou^¶^, Yan Dang, Xueping Huang, Chenyue Xu, Fang Xu, Xinyi Xu, Peng Li, Shutian Zhang, Haiyun Shi^*^, Jing Wu^*^

**S1 Fig. Rarefaction curve of samples (Shannon index).**

**S2 Fig. Rarefaction curve of samples (Good’s coverage).**

**S3 Fig. Relative abundance barplots of differential genus discovered by LEfSe analysis between IBD patients with anxiety and without anxiety or depression.**

**S4 Fig. Relative abundance barplots of differential genus discovered by LEfSe analysis between IBD patients with depression and without anxiety or depression.**

**S5 Fig. Relative abundance barplots of differential genus discovered by LEfSe analysis between IBD patients with and without anxiety and depression.**

**S6 Fig. Relative abundance barplots of differential genus discovered by spearman correlation analysis.**

**S7 Fig. Differential KEGG pathways discovered by multivariate linear model in IBD patients with anxiety.**

**S8 Fig. Differential KEGG pathways discovered by multivariate linear model in IBD patients with depression.**

**S9 Fig. Differential KEGG pathways discovered by multivariate linear model in IBD patients with both anxiety and depression.**

**S10 Fig. Sub-group analysis of IBD patients divided by disease activity and mental disorders. Alpha diversity described with Shannon index (A), PCoA plot (B) representing beta diversity, microbial community composition in (C) phylum, (D) family and (E) genus levels, LEfSe bar plot of differential genera (F), relative abundance heatmap of Tax4Fun2 functional annotations (Level 3) in remission (G)/active(I), differential analysis of the relative abundance of Tax4Fun2 functional annotations (Level 3) in remission (H)/active(J).** IBD - rem – NonAD, IBD patients in remission and without anxiety or depression. IBD - rem - A and/or D, IBD patients in remission and with anxiety and/or depression. IBD - act – NonAD, active IBD patients without anxiety or depression. IBD - act – A and/or D, active IBD patients with anxiety and/or depression.

**S1 Table. Data filtration and quality control information table.** RawPE, original PE reads. Combined, sequence of tags obtained by splicing. Qualified, the sequence of Raw Tags after filtering low quality and short length. Nochime, the sequence of Tags after filtering the chimeric, which is ultimately used for subsequent analysis, i.e. Effective Tags. Base, the number of bases of the final Effective Tags. AvgLen, average length of effective tags. Effective (%), the percentage of the number of Nochime to the number of rawPE.

**S2a-c Table PERMANOVA of IBD patients with mental disorders.**

**S3a-c Table. Differential ASVs of IBD patients with mental disorders (analysis performed by DESeq2).** ASVs, Amplicon Sequence Variants.

**S4a-c Table. Differential analysis of KEGG pathways in IBD patients with mental disorders.**

**S5 Table. Differential metabolites of IBD patients combined with anxiety.**

**S6 Table. Differential metabolites of IBD patients combined with depression.**

**S7 Table. Differential metabolites between IBD patients with or without anxiety and depression.**

**S8a Table. Baseline information of IBD patients with or without anxiety and or depression.**

**S8b Table. PERMANOVA of IBD patients with mental disorders.**

**S8c Table. Differential ASVs in IBD patients in remission with anxiety and/or depression (analysis performed by DESeq2).** ASVs, Amplicon Sequence Variants.

**S8d Table. Differential ASVs in active IBD patients with anxiety and/or depression (analysis performed by DESeq2).** ASVs, Amplicon Sequence Variants.

**S8e Table. Differential metabolites in IBD patients in remission with anxiety and/or depression.**

**S8f Table. Differential metabolites in active IBD patients with anxiety and/or depression.**
