# Supplementary figures and images for: Fecal microbiota composition and function are associated with anxiety and depression in patients with inflammatory bowel disease
Source: PLoS One. 2025 Dec 19;20(12):e0337941. doi: 10.1371/journal.pone.0337941 (PMC12716764; doi:10.1371/journal.pone.0337941)

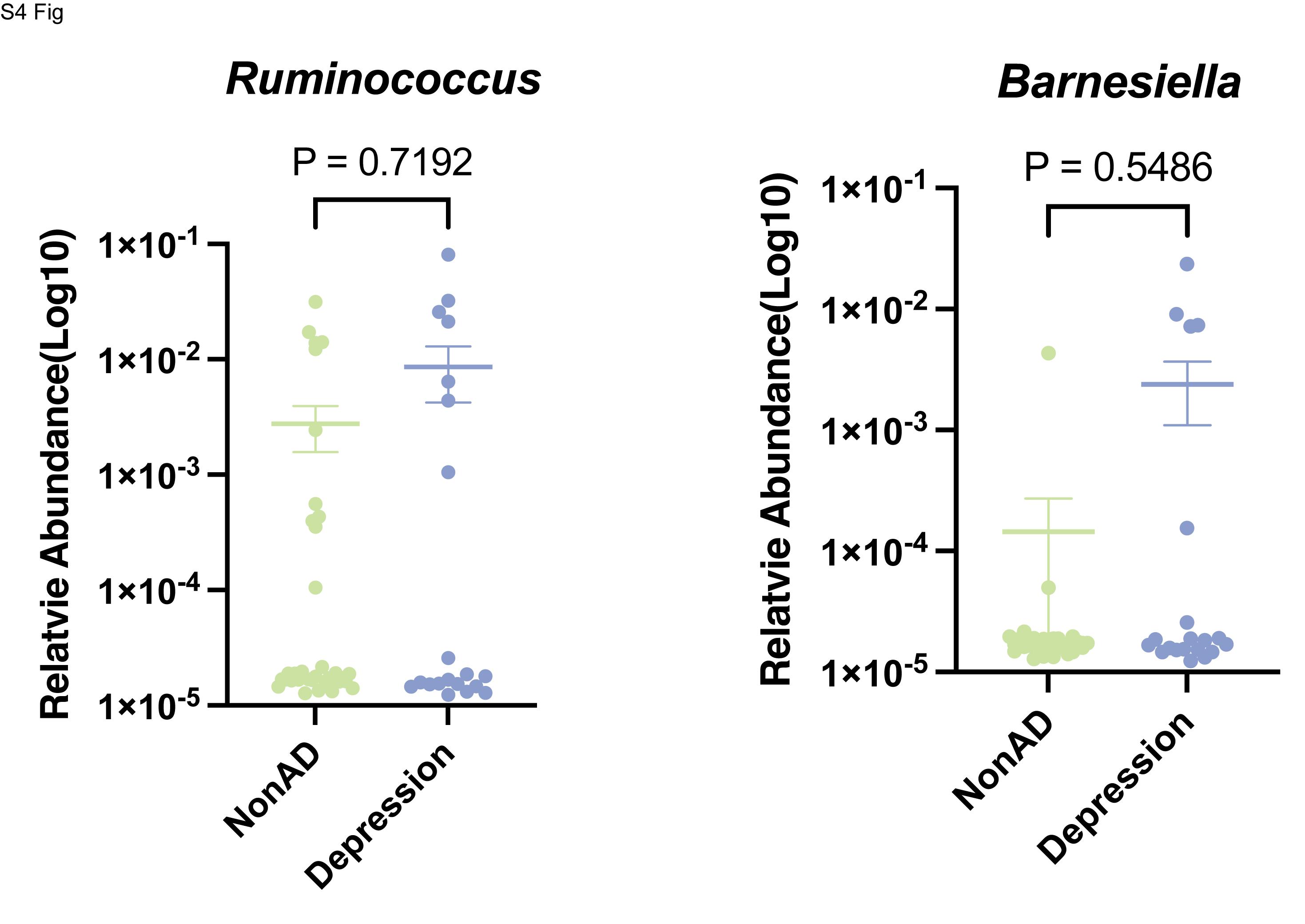

Supplement: S1 File — (ZIP) [file pone.0337941.s001.zip › supplementary materials/S4_Fig.tif]

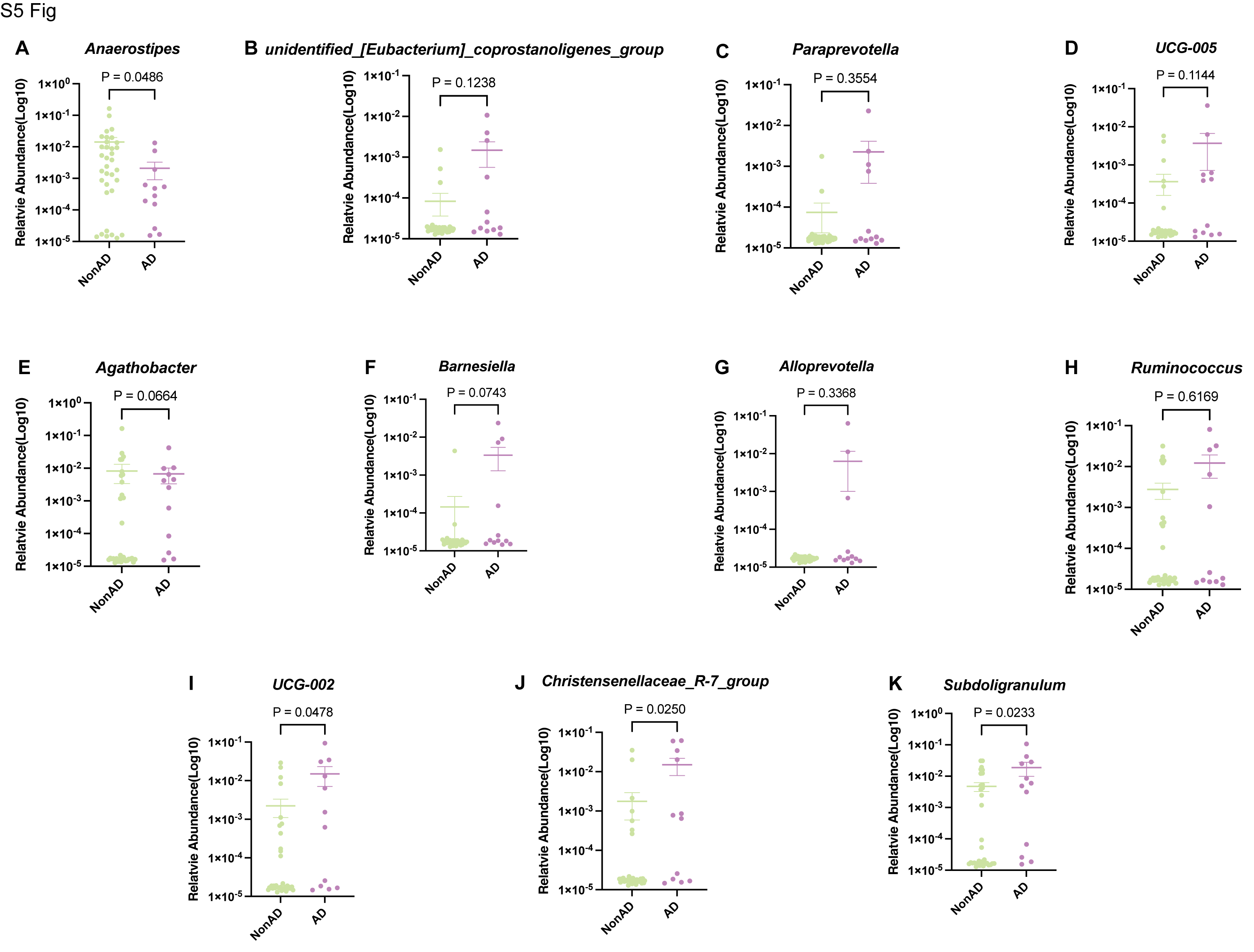

Supplement: S1 File — (ZIP) [file pone.0337941.s001.zip › supplementary materials/S5_Fig.tif]

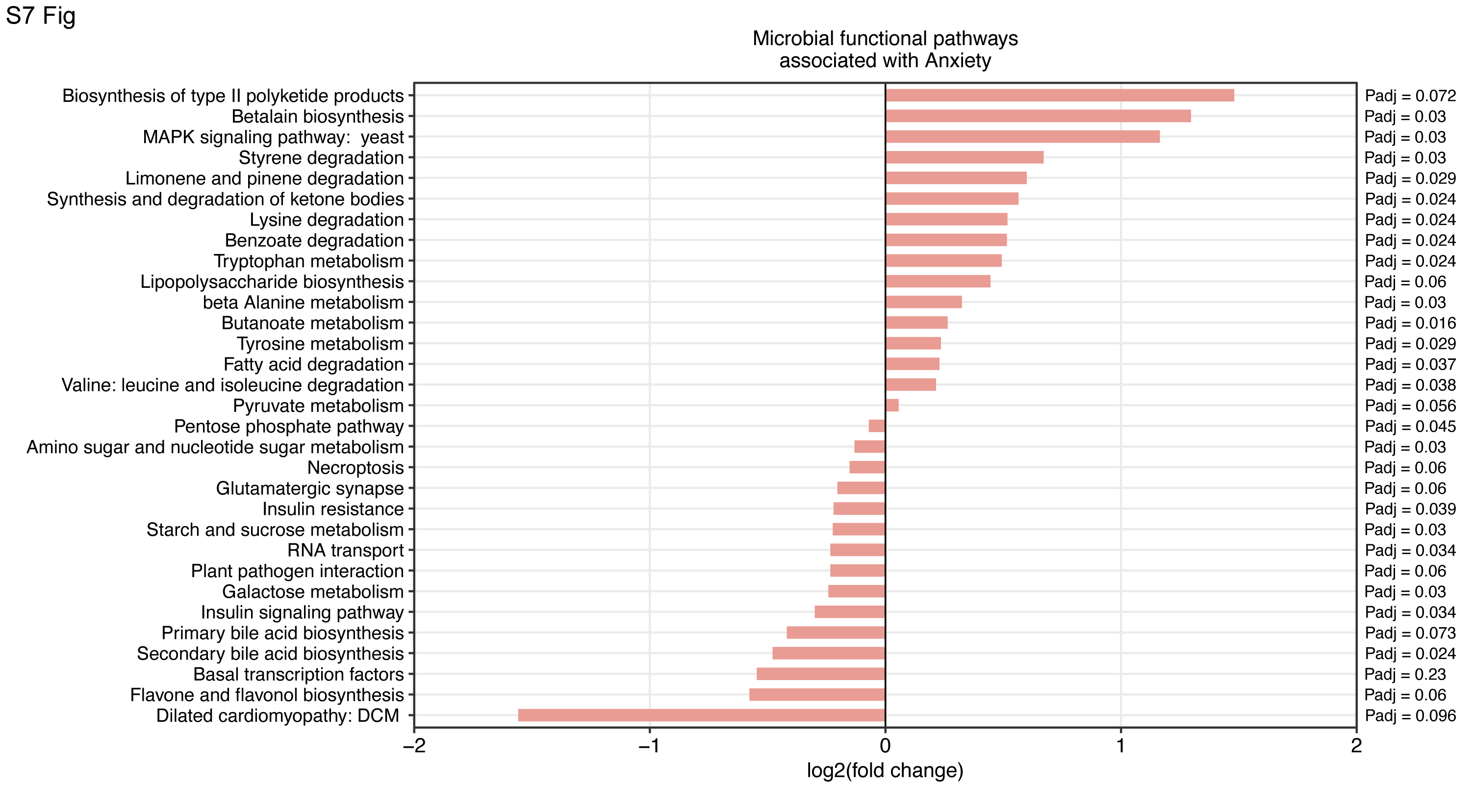

Supplement: S1 File — (ZIP) [file pone.0337941.s001.zip › supplementary materials/S7_Fig.tif]

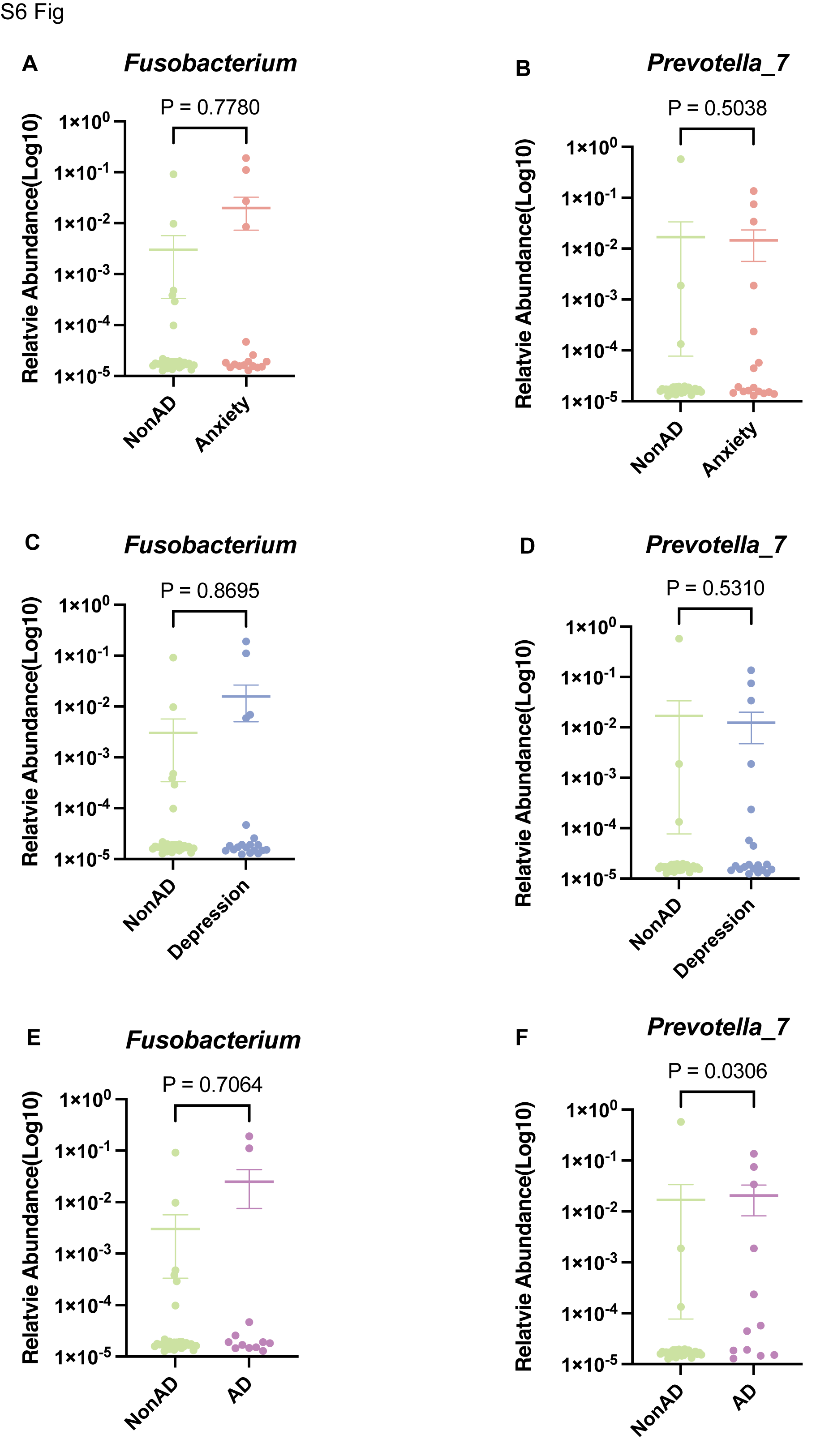

Supplement: S1 File — (ZIP) [file pone.0337941.s001.zip › supplementary materials/S6_Fig.tif]

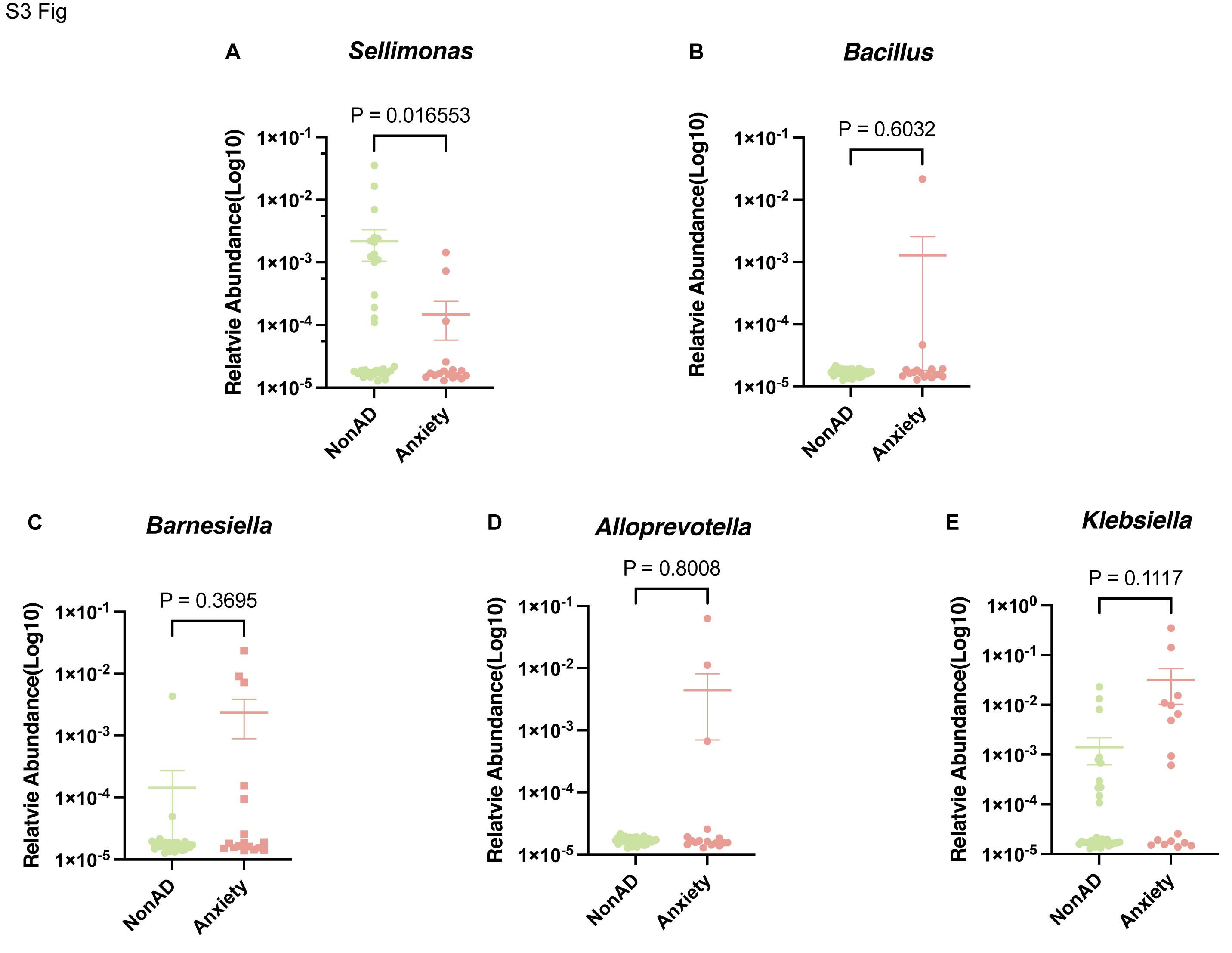

Supplement: S1 File — (ZIP) [file pone.0337941.s001.zip › supplementary materials/S3_Fig.tif]

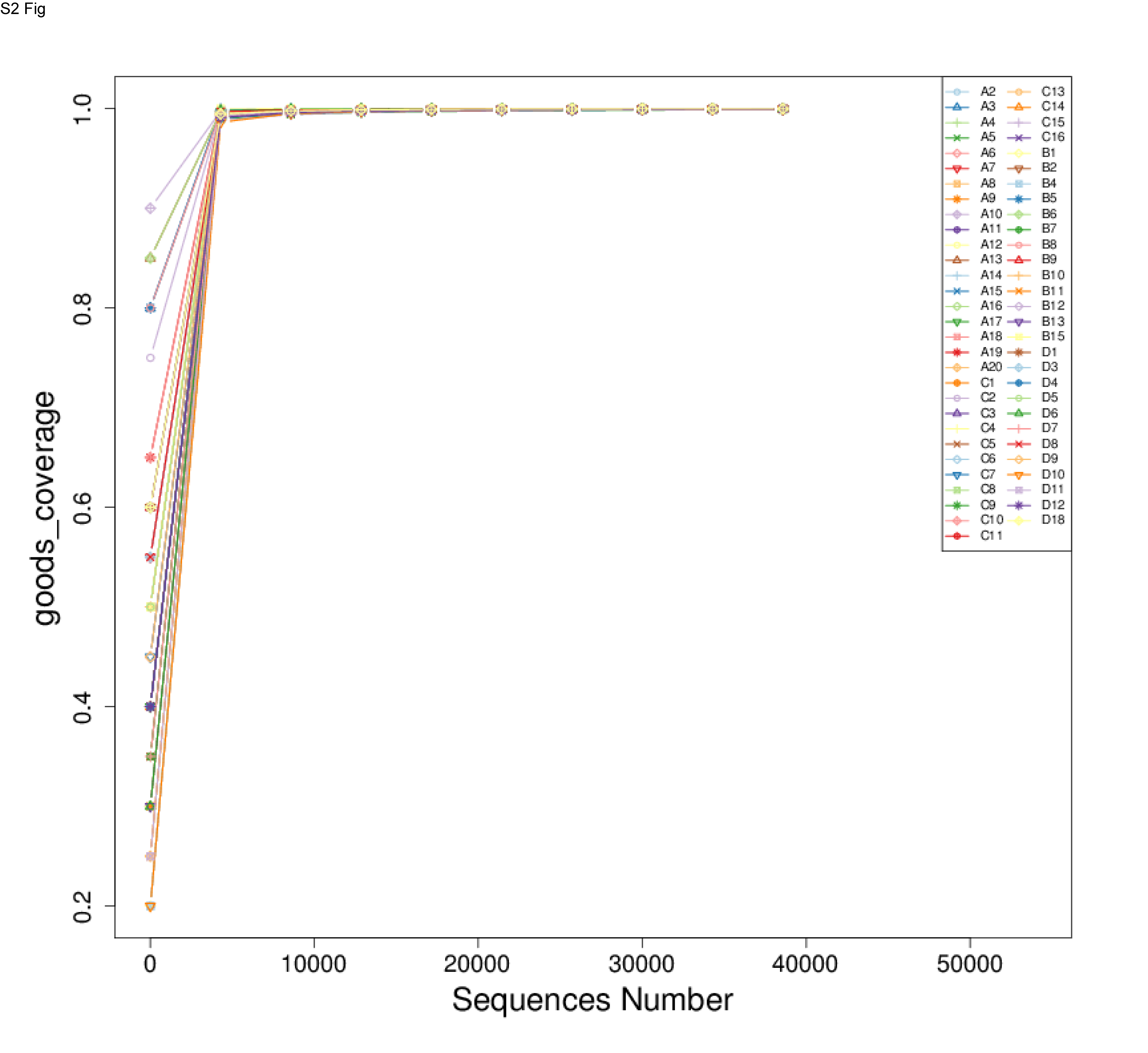

Supplement: S1 File — (ZIP) [file pone.0337941.s001.zip › supplementary materials/S2_Fig.tif]

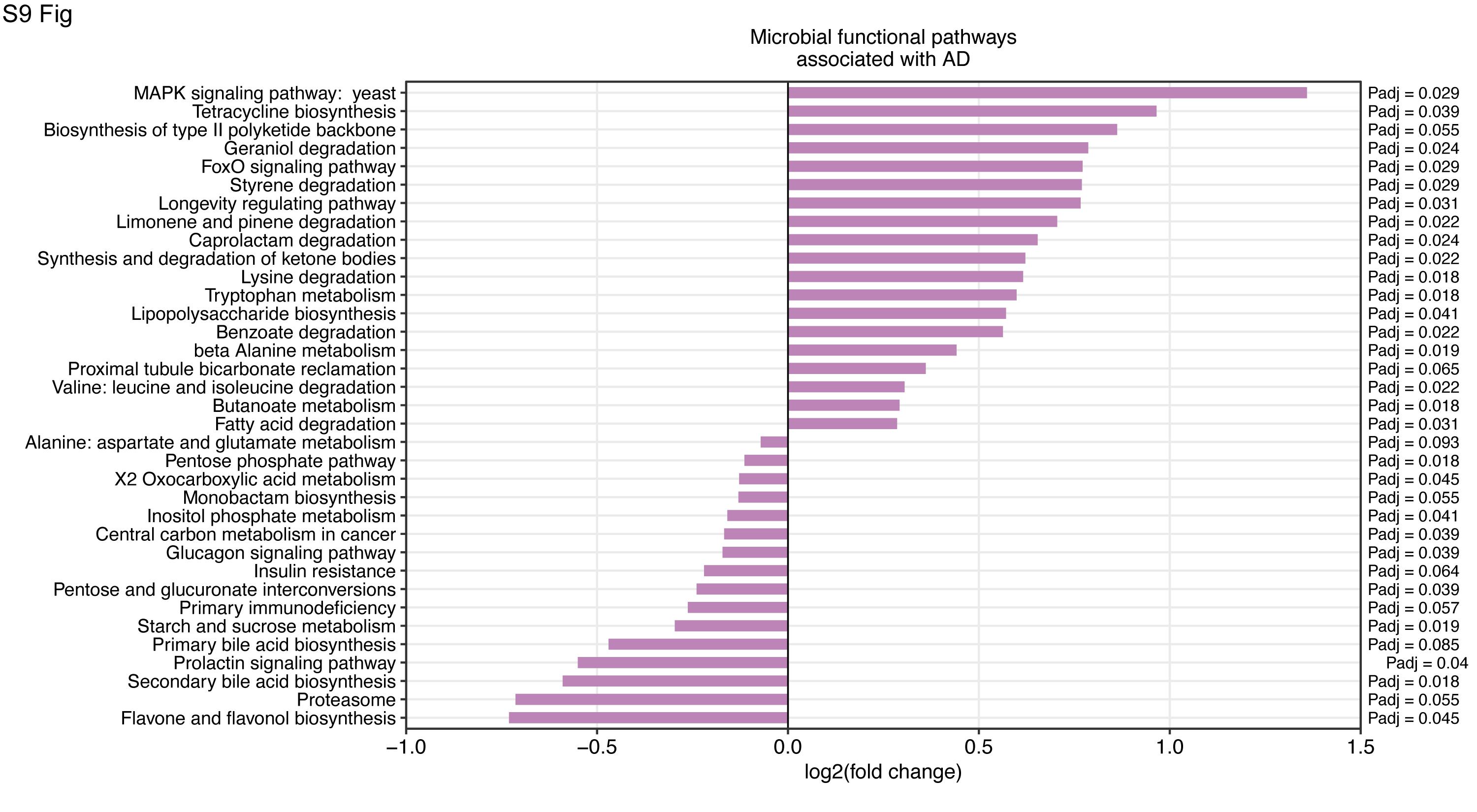

Supplement: S1 File — (ZIP) [file pone.0337941.s001.zip › supplementary materials/S9_Fig.tif]

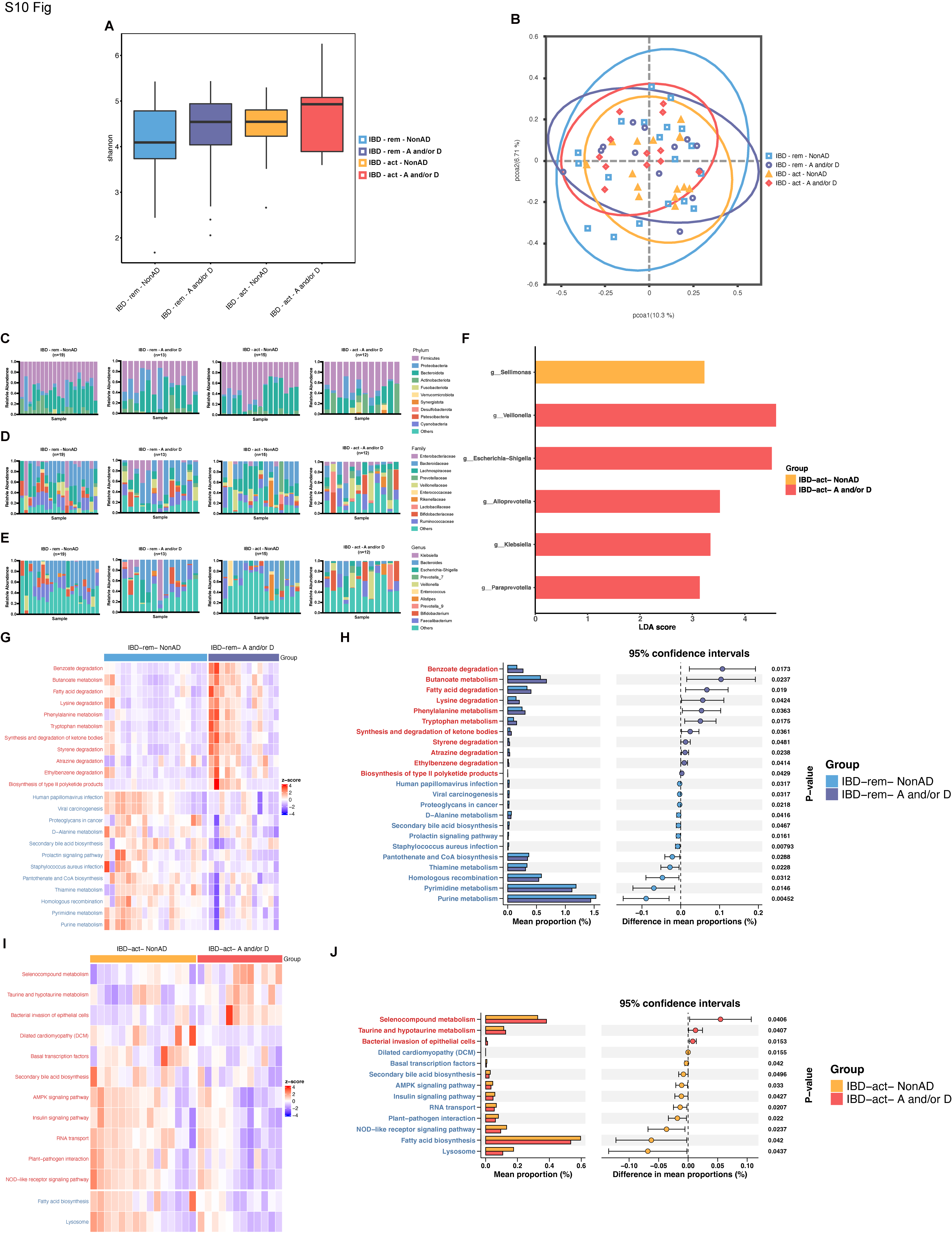

Supplement: S1 File — (ZIP) [file pone.0337941.s001.zip › supplementary materials/S10_Fig.tif]

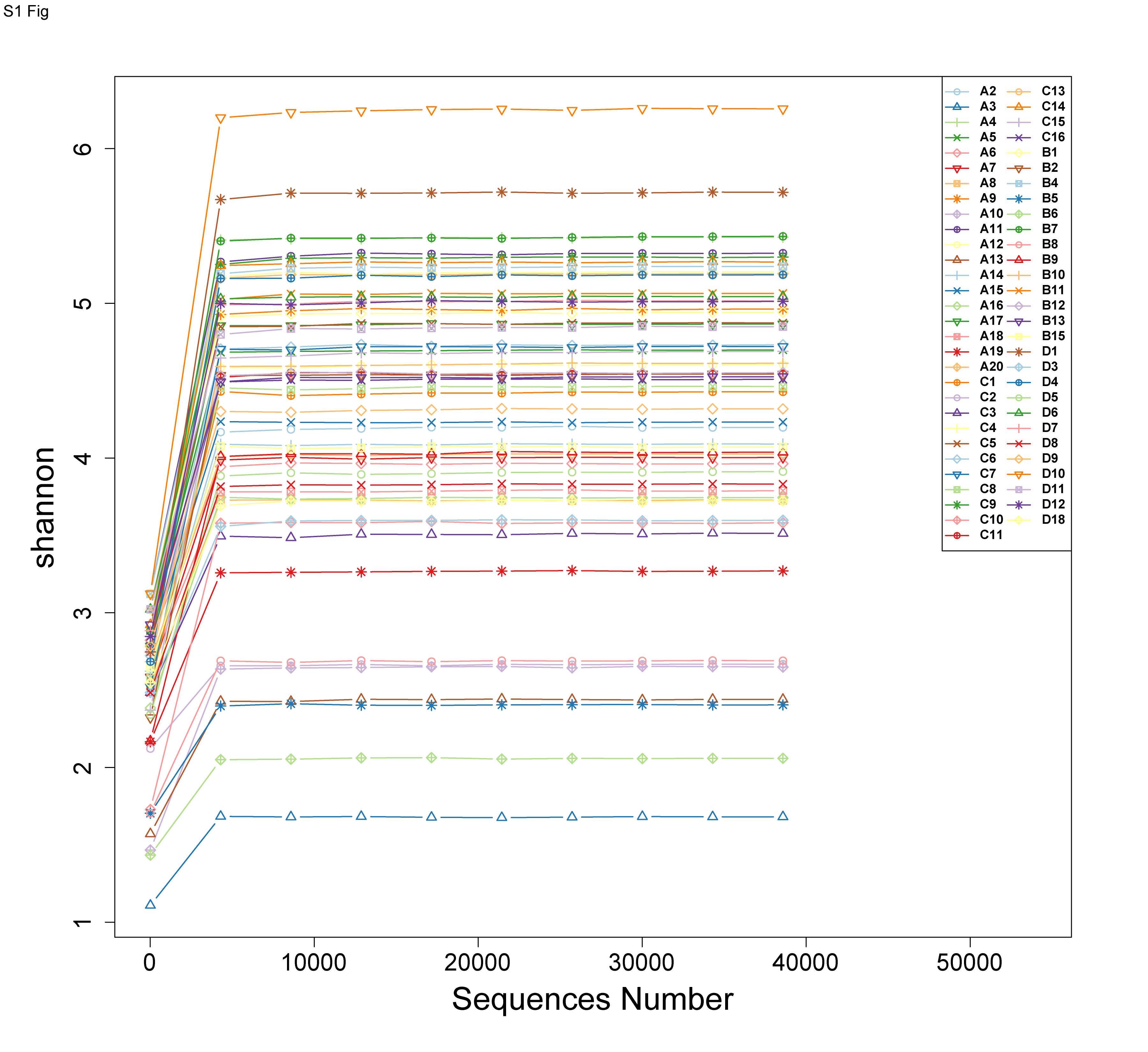

Supplement: S1 File — (ZIP) [file pone.0337941.s001.zip › supplementary materials/S1_Fig.tif]

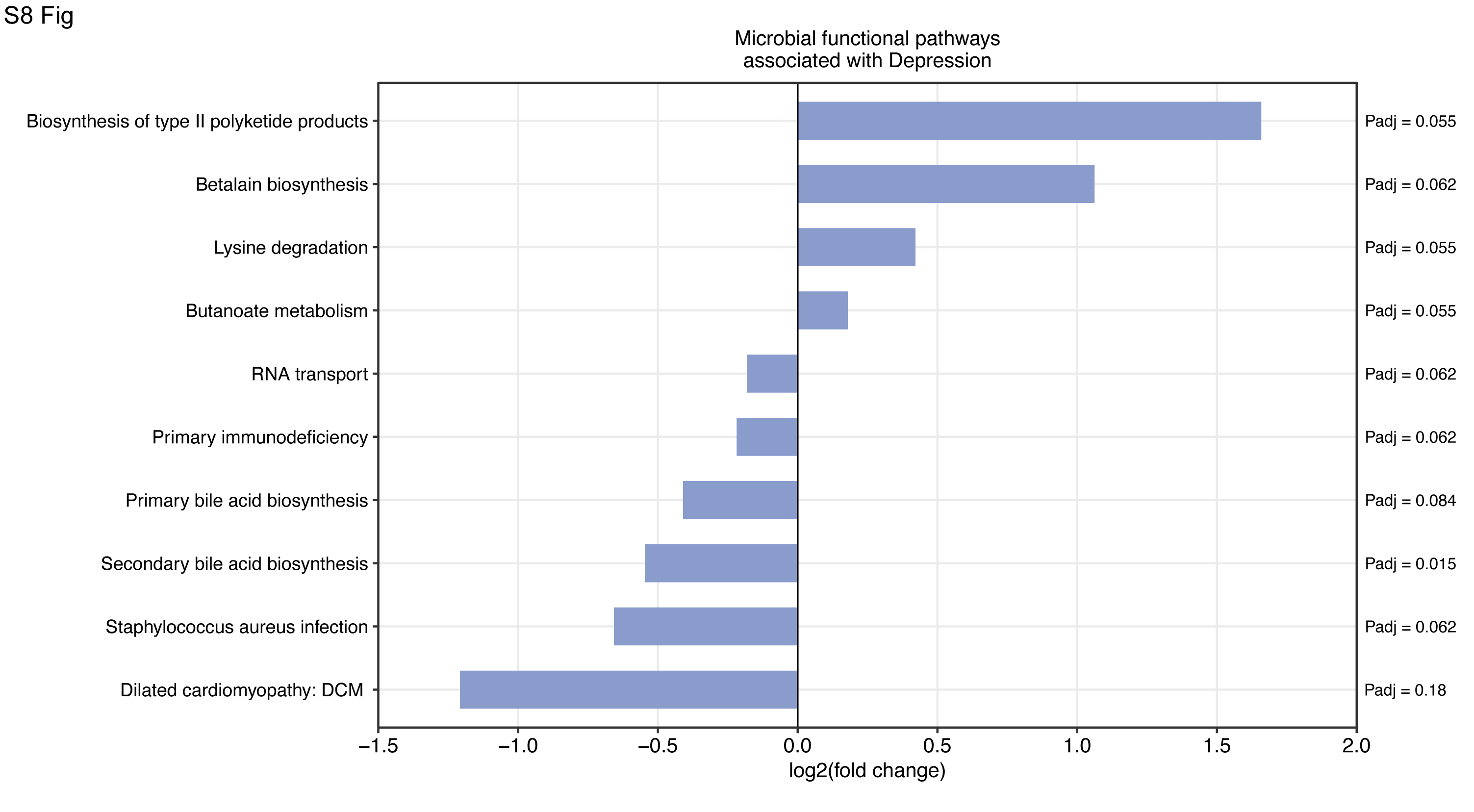

Supplement: S1 File — (ZIP) [file pone.0337941.s001.zip › supplementary materials/S8_Fig.tif]
